# Combination of Infliximab and High-Dose Intravenous Immunoglobulin for Toxic Epidermal Necrolysis: Successful Treatment of an Elderly Patient

**DOI:** 10.1155/2012/915314

**Published:** 2012-10-09

**Authors:** Konstantinos Patmanidis, Anastasios Sidiras, Konstantinos Dolianitis, Dimitrios Simelidis, Christos Solomonidis, Georgios Gaitanis, Ioannis D. Bassukas

**Affiliations:** ^1^Department of Skin and Venereal Diseases, University of Ioannina Medical School, 45110 Ioannina, Greece; ^2^Department of Internal Medicine, Kozani General Hospital, 50100 Kozani, Greece; ^3^Dermatology Service, Kozani General Hospital, 50100 Kozani, Greece

## Abstract

Toxic epidermal necrolysis (TEN) is a rare, severe cutaneous adverse drug reaction with average mortality 25–35%, especially among elderly multimorbid patients. Established therapeutic guidelines do not exist and controversies underlie many of the presently suggested treatment regimens. Herein we present the use of the recently described combination scheme of methylprednisolone (500 mg methylprednisolone bolus i.v.) followed by infliximab (5 mg/kg i.v.) and high-dose intravenous immunoglobulin (2 g/kg over 5 days) to treat an elderly, 74-year-old female patient with TEN (SCORTEN 3) within the premises of a district hospital. Already from the second day of hospitalization the skin condition markedly stabilized and the patient's status improved rapidly thereafter. She was discharged after 19 days in stationary care in excellent general condition and remained without any sequels 9 months afterwards. The present paper further supports the feasibility, efficacy, and safety of the proposed combination modality for the treatment of elderly patients with TEN, a population susceptible to more severe TEN.

## 1. Introduction

Toxic epidermal necrolysis (TEN) is a rare, severe cutaneous adverse drug reaction presenting with widespread necrosis of epidermal keratinocytes that leads to critical, potentially life-threatening skin insufficiency and dysfunction, associated with significant morbidity and mortality [1, 2]. The average mortality of this condition is 25–35% but it can be much higher among elderly multimorbid patients [2]. Furthermore, more than 50% of TEN survivors suffer from long-term sequels [2]. Established therapeutic guidelines do not exist and controversies underlie many of the presently suggested treatment regimens [1, 2]. Current data are indicative for the use of high-dose intravenous immunoglobulin (hIVIg), whereas inconclusive evidence exists for the addition of corticosteroids [2, 3]. 

Herein, we describe the effective use of the recently described combinational therapeutic protocol of methylprednisolone, tumor necrosis factor alpha (TNFa) blockage with infliximab and high-dose intravenous immunoglobulin for TEN^4^ in an elderly patient within the premises of a district hospital.

## 2. Case Report

A 74-year-old female patient was admitted to the Internal Medicine Department of the public Kozani General Hospital with extended skin detachment of 24-hour duration. For the past 7 days she had received trimethoprim-sulfamethoxazole (960 mg twice daily) for a lower urinary tract infection. 

**Figure 1:**
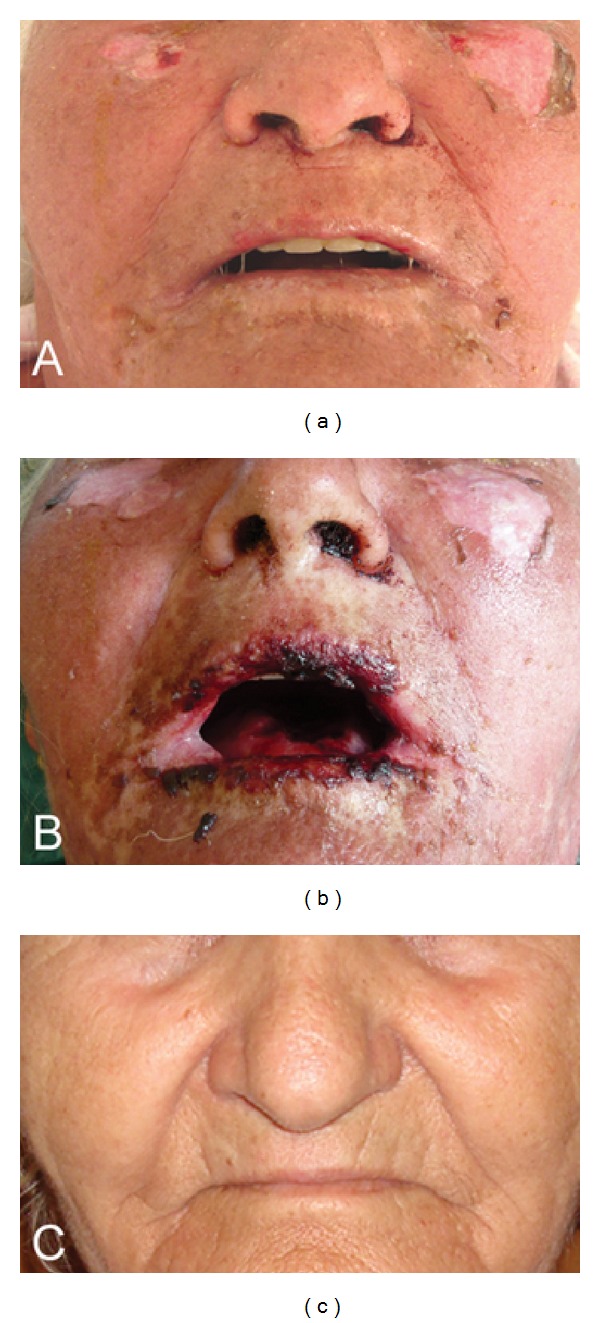
Detail of patient's face. Panel (a): diffuse erythema and beginning of detachment of the epidermis at admission. Panel (b): at day 4 of hospitalization presence of hemorrhagic stomatitis and cheilitis, however with already arrested disease progression in the skin. Panel (c): state of complete recovery at 9-month follow-up appointment.

On admission confluent erythema and widespread epidermal necroses (~25% body surface) were present that affected face, trunk, and extremities including palms and soles, with a positive Nikolsky's sign (Figure 1). Mouth erosions and enema of conjunctivae were also present. Her vitals included 140/65 mmHg blood pressure, 78 pulses/min, and temperature 38.5°C. Her medical history was remarkable for arterial hypertension on perindopril/indapamide, nephrolithiasis treated with citric acid/monosodium citrate *per os* and anxiety on bromazepam and alprazolam. She was on these medications without any alterations for the previous 6 months. The laboratory workout (Table 1) revealed white blood count 3.72 × 10^3^ cells/mL (75% neutrophils, 20% lymphocytes, 3% monocytes); hemoglobin: 10.7 g/dL; hematocrit: 32.7%; platelets: 221 × 10^3^ cells/mL; C-reactive protein: 10.6 mg/dL; blood urea nitrogen: 107 mg/dL; serum creatinin: 1.91 mg/dL; alkaline phosphatase: 40 ng/mL; serum glucose: 208 mg/dL; aspartate aminotransferase: 92 U/L; alanine aminotransferase: 177 U/L; bicarbonates: 20.1 mEq/L; Na^+^: 131 mEq/L; K^+^: 4.08 mEq/L; total serum protein: 6.3 g/dL. The constellation of clinical and laboratory data sets the diagnosis of TEN. Her SCORTEN index^2^ was 3 (age > 40 years old, blood urea nitrogen > 28 mg/dL, body surface involvement > 10%) which corresponds to an expected mortality risk of 35.8%.

Immediately after the establishment of diagnosis, treatment was initiated *in loco* according to a recent therapeutic proposal [4]. She was not transferred to a reference center as she strongly wished to be nursed nearby her residence. Additionally her unstable condition was considered as a relative hindrance for this. The patient received 500 mg methylprednisolone bolus i.v. followed by 5 mg/kg i.v. infliximab. High-dose intravenous immunoglobulin (2 g/kg) was initiated the same day and was given over the next 5 days. She was also treated with appropriate supportive measures, including careful monitoring of liquid and electrolytes equilibrium and wound care. Already from the second day of hospitalization the skin condition markedly stabilized and the patient's status improved rapidly thereafter (Figure 1). The patient was discharged after 19 days in stationary care in excellent general condition and remained without any sequels 9 months afterwards.

## 3. Discussion

To the best of our knowledge this is the fourth patient treated with the present protocol. Extensive epidermal keratinocyte cell death via cytotoxic T-cell stimulation, activation of Fas-Fas ligand interactions, and increased TNFa levels locally in the skin and in the circulation underlie the pathogenesis of TEN. It is suggested that the present combination modality efficiently arrests the detrimental progression of this condition through targeted modification of the above pathophysiologic aberrations [4]. Pharmacologic targeting of increased TNFa levels in TEN patients seems to be a promising emerging treatment strategy [4]. However, designing trials to prove the efficacy of a therapeutic approach in a rare and serious disease as is TEN is not without any pitfalls. Thus, a study aiming at evaluating the efficacy of thalidomide was prematurely terminated because of failing effectiveness [5] while another trial on the efficacy of infliximab was also prematurely terminated due to inadequate recruitment (NCT00372723: http://www.clinicaltrials.gov/; last accessed: May 31, 2012). 

The present paper further supports the feasibility, efficacy, and safety of the proposed combination modality for elderly patients with TEN [4]. This population can be considered the target of such therapeutic approaches due to the fact that it is susceptible to the development of TEN, as polypharmacy increases the risk and the presence of comorbidities contributes to the overall mortality of this condition. 

## Figures and Tables

**Table 1:** Core physical and laboratory data of the patient at admission. Parameters included in the calculation of the SCORTEN index are indicated in bold; parameters adding to SCORTEN in the present case are indicated in italics (SCORTEN index = 3).

	Patient's findings	SCORTEN index
Physical findings at admission		
*Age (years) *	74	***〈*** **+** ***〉***
*Body surface affected (%) *	25	***〈*** **+** ***〉***
Pulses/min	78	〈−〉
Malignancy (history)	No	〈−〉
Temperature (°C)	38.5	
Blood pressure systolic/diastolic (mmHg)	140/65	
Laboratory findings at admission		
Hematocrit (%)	32.7	
Hemoglobin (g/dL)	10.7	
White blood cell count/mL	3.72 × 10^3^	
Platelets/mL	221 × 10^3^	
Erythrocyte sedimentation rate (mm/h)	17	
C-reactive protein (mg/dL)	10.6	
Glucose (mg/dL)	208	〈−〉
*Urea nitrogen (mg/dL) *	107	***〈*** **+** ***〉***
Creatinine (mg/dL)	1.91	
Sodium (mEq/L)	131	
Potassium (mEq/L)	4.08	
Bicarbonate (mEq/L)	20.1	〈−〉
Total serum protein (g/dL)	6.3	
Alanine aminotransferase (U/L)	177	
Aspartate aminotransferase (U/L)	92	
Alkaline phosphatase (ng/mL)	40	
